# Multi‐Targeting DKK1 and LRP6 Prevents Bone Loss and Improves Fracture Resistance in Multiple Myeloma

**DOI:** 10.1002/jbmr.4809

**Published:** 2023-05-25

**Authors:** Marija K. Simic, Sindhu T. Mohanty, Ya Xiao, Tegan L. Cheng, Victoria E. Taylor, Olga Charlat, Peter I. Croucher, Michelle M. McDonald

**Affiliations:** ^1^ Skeletal Diseases Program Garvan Institute of Medical Research Darlinghurst NSW Australia; ^2^ St Vincent's Clinical Campus, School of Clinical Medicine University of New South Wales Kensington NSW Australia; ^3^ Centre for Children's Bone and Musculoskeletal Health The Children's Hospital at Westmead Westmead NSW Australia; ^4^ Novartis Institutes for Biomedical Research Cambridge MA USA; ^5^ School of Medical Science, Faculty of Medicine and Health The University of Sydney Sydney NSW Australia

**Keywords:** WNT, LRP6, DKK1, MYELOMA, BONE

## Abstract

An imbalance between bone resorption and bone formation underlies the devastating osteolytic lesions and subsequent fractures seen in more than 90% of multiple myeloma (MM) patients. Currently, Wnt‐targeted therapeutic agents that prevent soluble antagonists of the Wnt signaling pathway, sclerostin (SOST) and dickkopf‐1 (DKK1), have been shown to prevent bone loss and improve bone strength in preclinical models of MM. In this study, we show increasing Wnt signaling via a novel anti–low‐density lipoprotein receptor‐related protein 6 (LRP6) antibody, which potentiates Wnt1‐class ligand signaling through binding the Wnt receptor LRP6, prevented the development of myeloma‐induced bone loss primarily through preventing bone resorption. When combined with an agent targeting the soluble Wnt antagonist DKK1, we showed more robust improvements in bone structure than anti‐LRP6 treatment alone. Micro–computed tomography (μCT) analysis demonstrated substantial increases in trabecular bone volume in naïve mice given the anti‐LRP6/DKK1 combination treatment strategy compared to control agents. Mice injected with 5TGM1eGFP murine myeloma cells had significant reductions in trabecular bone volume compared to naïve controls. The anti‐LRP6/DKK1 combination strategy significantly improved bone volume in 5TGM1‐bearing mice by 111%, which was also superior to anti‐LRP6 single treatment; with similar bone structural changes observed within L_4_ lumbar vertebrae. Consequently, this combination strategy significantly improved resistance to fracture in lumbar vertebrae in 5TGM1‐bearing mice compared to their controls, providing greater protection against fracture compared to anti‐LRP6 antibody alone. Interestingly, these improvements in bone volume were primarily due to reduced bone resorption, with significant reductions in osteoclast numbers and osteoclast surface per bone surface demonstrated in 5TGM1‐bearing mice treated with the anti‐LRP6/DKK1 combination strategy. Importantly, Wnt stimulation with either single or combined Wnt‐targeted agents did not exacerbate tumor activity. This work provides a novel approach of targeting both membrane‐bound and soluble Wnt pathway components to provide superior skeletal outcomes in patients with multiple myeloma and other bone destructive cancers. © 2023 The Authors. *Journal of Bone and Mineral Research* published by Wiley Periodicals LLC on behalf of American Society for Bone and Mineral Research (ASBMR).

## Introduction

Bone metastasis is a common occurrence in many malignancies, including breast and prostate cancers, that can lead to significant bone destruction and consequently fractures.^(^ ^1^, ^2^ ^)^ In particular, multiple myeloma (MM), a B‐cell malignancy that develops within the bone marrow, has the highest prevalence of bone involvement compared to other malignancies. More than 90% of myeloma patients develop bone disease causing severe bone destruction and debilitating pain, which significantly impacts on their quality of life.^(^ ^3^ ^)^ This myeloma‐induced bone destruction arises form an imbalance in the bone remodeling process with increased osteoclastic bone resorption and reduced osteoblastic bone formation.^(^ ^3^, ^4^ ^)^ Anti‐resorptive agents such as the bisphosphonate zoledronic acid (ZA), and denosumab, a monoclonal antibody that targets the receptor activator of nuclear κβ ligand (RANKL), are both currently used as the gold standard approach for preventing further bone destruction from occurring in patients.^(^ ^5^, ^6^ ^)^ Although the incidence of skeletal‐related events is reduced with these agents, they do not stimulate new bone formation, and many patients continue to fracture. Thus, novel therapeutic strategies to treat myeloma‐induced bone disease should also stimulate osteoblastic bone formation to overcome the debilitating skeletal pathology in patients with MM.

The canonical Wnt/β‐catenin signaling pathway and Wnt ligands have been associated with numerous bone metabolic disorders and diseases,^(^ ^7^, ^8^ ^)^ and also play an important role in osteoblastogenesis and the regulation of bone metabolism.^(^ ^9^, ^10^ ^)^ Soluble Wnt antagonists, such as dickkopf‐1 (DKK1) and sclerostin (SOST), are critical components of this pathway and inhibit bone formation when secreted locally by osteoblasts (OBs), bone marrow stromal cells (BMSCs), and osteocytes. Indeed, neutralizing antibodies to these antagonists have strong bone anabolic potential, with romosozumab (anti‐SOST) approved for clinical use to increase bone mass in osteoporosis and other low bone mass indications.^(^ ^11^, ^12^ ^)^

In addition to targeting soluble Wnt antagonists such as DKK1 and SOST, a novel approach to stimulate Wnt signaling, and hence bone formation, is targeting the low‐density lipoprotein receptor‐related protein 5/6 (LRP5/6) receptor, to which Wnt1 and Wnt3a class ligands bind and stimulate Wnt signaling.^(^ ^8^ ^)^ Studies have looked into the structure and pivotal role of the receptor in activating the Wnt signaling pathway and its importance in bone development.^(^ ^13^, ^14^ ^)^ Moreover, various antibodies targeting specific regions of the LRP6 receptor have been developed to stimulate Wnt signaling.^(^ ^15^, ^16^ ^)^ In fact, potentiation of LRP6‐mediated Wnt1 class signaling while blocking Wnt3a class binding led to greater increases in bone mass in mice than an antibody that potentiates Wnt3a class signaling.^(^ ^17^ ^)^ The cellular and molecular mechanisms underlying this improvement in bone mass and the potential for LRP6‐targeting agents to prevent myeloma‐induced osteolytic disease remain unknown.

To address this, we examined the impact of a novel anti‐LRP6 antibody that potentiates LRP6‐mediated Wnt1 class signaling in normal and myeloma‐burdened bone conditions. We first determined the cellular mechanisms for this improvement in bone structure with anti‐LRP6, and whether this LRP6‐targeted agent prevents the development of osteolytic bone loss utilizing the 5TGM1 murine model of MM. Targeting multiple components of the Wnt/β‐catenin signaling has been previously explored, revealing additive effects on bone mass. Combination of the soluble antagonists DKK1 and SOST inhibition using a bispecific antibody strategy provided superior increases in bone mass and fracture repair in rodents compared to single treatment approaches.^(^ ^18^ ^)^ We therefore applied this approach in our 5TGM1 murine model of myeloma, targeting both the LRP6 receptor and the soluble Wnt antagonist DKK1. DKK1 is secreted by MM cells and therefore elevated local levels of DKK1 has been confirmed in the pathogenesis of MM‐induced bone loss.^(^ ^19^, ^20^, ^21^ ^)^ Therapeutic inhibition of DKK1 increased bone formation and prevented bone loss in experimental models of myeloma.^(^ ^22^, ^23^ ^)^ Therefore, we hypothesized that this novel combination receptor/antagonist targeted approach would be superior in preventing MM‐induced bone disease.

We show that anti‐LRP6 could be of therapeutic benefit to prevent myeloma‐induced bone loss, but superior protection against bone loss and reduced strength was demonstrated when used in combination with anti‐DKK1. Overall, we provide evidence that therapeutically targeting multiple components of the Wnt/β‐catenin signaling pathway may reduce skeletal damage and fracture in patients suffering from bone destructive cancers.

## Materials and Methods

### Myeloma cell lines

Murine 5TGM1‐enhanced green fluorescent protein (95TGM1eGFP) myeloma cells were cultured as described.^(^ ^24^, ^25^ ^)^ In summary, 5TGM1eGFP myeloma cells were cultured in RPMI‐1640 media supplemented with 10% fetal calf serum (FCS) and 1% penicillin–streptomycin (P/S) and were incubated in 5% CO_2_ at 37°C.

### Interventions

Anti‐LRP6, anti‐DKK1 and their respective control (immunoglobulin G [IgG] isotype) antibodies were all generated by Novartis Pharma in phosphate buffered saline (PBS), at a dose of 10 mg/kg intravenously (i.v.) as described^(^ ^16^ ^)^ and is near the maximum tolerable dose. The dose rate for anti‐DKK1 have been reported elsewhere.^(^ ^23^ ^)^ No adverse effects of any agent were noted in mice.

### Experimental mice

Animal experiments were performed in accordance with approved protocols from the Garvan Institute/St Vincent's Hospital Animal Ethics Committee (ARA 18/08) and the Australian Code of Practice for the Care and Use of Animals for Scientific Purposes. Female C57BLKalwRij mice were purchased from the Australian BioResources facility and were housed in the biological testing facility at the Garvan Institute of Medical Research. Upon arrival, all mice were given 3 days to acclimatize, and standard chow food and water were provided *ad libitum*. All mice were entered into their respective experiments aged 6–8 weeks. For all in vivo experiments, group sizes were determined based upon previous experience with each model system. This included studies of the effect of bone anabolic drugs in these models, in which power calculations were conducted to estimate sample size. Consequently, 6–8 mice were allocated to each group or as otherwise stated in the figure legends.

**Fig. 1:**
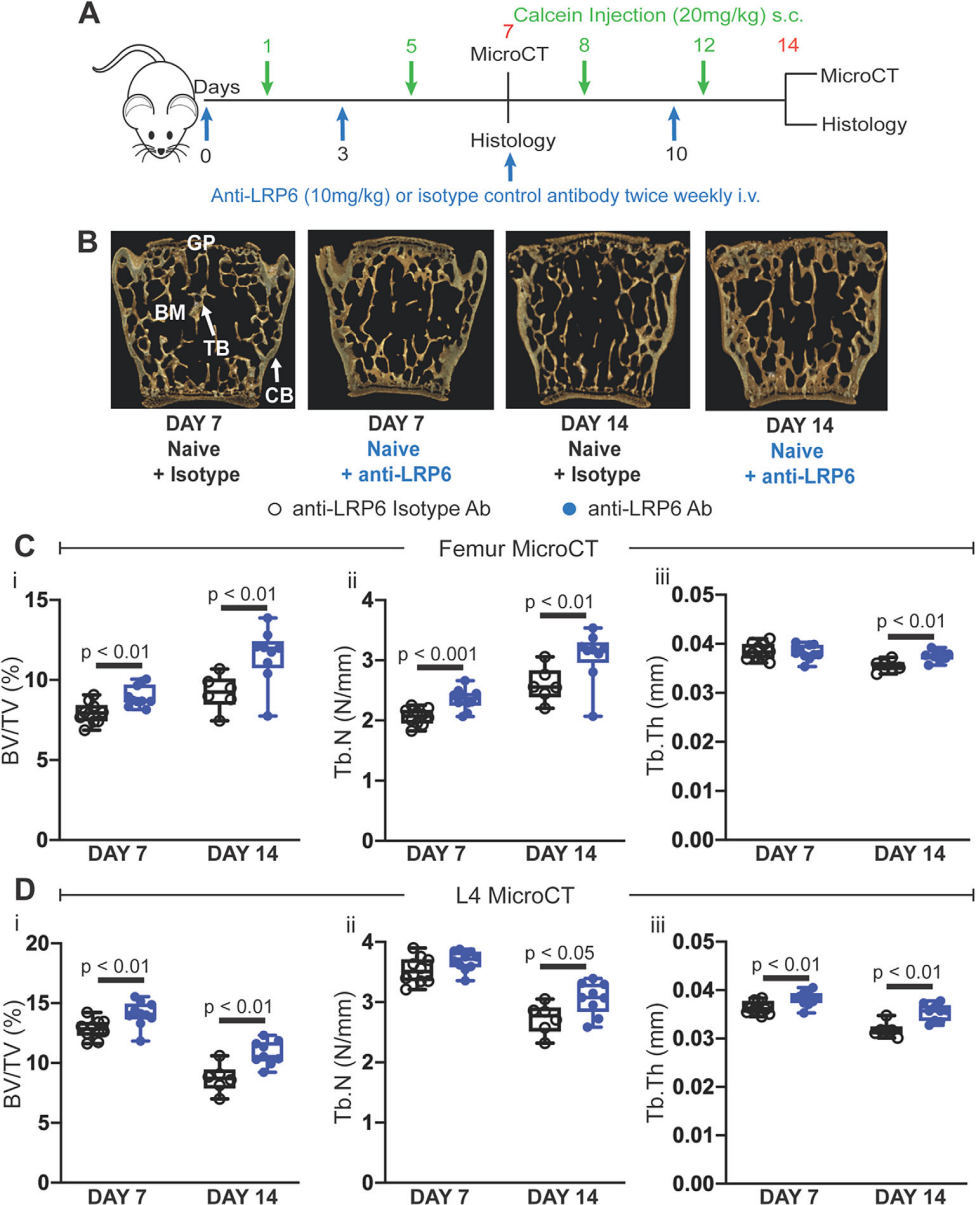
Anti‐LRP6 antibody increased trabecular bone volume in femurs and L_4_ lumbar vertebrae in naïve mice. (*A*) Experimental design and timeline, including antibody treatment duration, fluorochrome labels, and time points where bone tissue were harvested. (*B*) 3D representative images of L_4_ lumbar vertebrae from each treatment group (isotype or anti‐LRP6 Ab) harvested at their respective time points (day 7 or 14). (*C*) μCT‐derived trabecular bone volume fraction (i, BV/TV, %), trabecular number (ii, Tb.N, N/mm), and trabecular thickness (iii, Tb.Th, mm) in the distal femoral metaphysis in all treatment groups at their respective time points. (*D*) μCT‐derived trabecular (i) BV/TV (%), (ii) Tb.N (N/mm), and (iii) Tb.Th (mm) in lumbar L_4_ vertebrae harvested from naïve mice treated with isotype or anti‐LRP6 Ab for 7 or 14 days. Results plotted as mean ± SD. Ab = antibody; BM = bone marrow; CB = cortical bone; GP = growth plate; i.v. = intravenous; s.c. = subcutaneous; TB = trabecular bone.

To determine the mechanism of action of anti‐LRP6 alone in naïve (non‐tumor bearing) mice, animals were randomly allocated to treatment groups. Anti‐LRP6 antibody or its isotype were administered twice weekly i.v. (10 mg/kg; Novartis Pharma, Cambridge, MA, USA) from day 1. At specific timepoints (Fig. 1*A*), mice were culled at days 7 or 14 where tissues were harvested and went to their respective experiments. For the 5TGM1 studies, female mice aged 6–8 weeks were injected via the tail vein with 2 × 10^6^ 5TGM1eGFP cells (hereby referred to as 5TGM1‐bearing), and mice were randomly allocated to treatment groups. Treatment with anti‐LRP6 alone or the anti‐LRP6/DKK1 combination strategies with their respective isotypes began the day following myeloma cell injection.

Mice were euthanized at 7 or 14 days (naïve mice) or 21 days (naïve and 5TGM1‐bearing mice) following treatment administration. Femurs were harvested and fixed in 4% paraformaldehyde in PBS for 24 hours and stored in 70% ethanol for micro–computed tomography (μCT) and histological analysis. Left femurs were decalcified for paraffin histology, while right femurs were processed for resin histology as reported elsewhere.^(^ ^24^ ^)^ L_4_ vertebrae were wrapped in saline‐soaked gauze and stored at −70°C for μCT analysis and biomechanical compression testing. For 5TGM1 studies, spleen and tibia were harvested for fluorescence activated cell sorting (FACS) analysis.

### Flow cytometry analysis of green fluorescence protein–labeled 5TGM1 myeloma cells in bone marrow and spleen

For 5TGM1 studies, bone marrow of tibias was flushed with 2% FCS in PBS following removal of proximal and distal ends. Samples were cut longitudinally; bone fragments were scraped, and all components were then flushed out with 2% FCS in PBS using a 23G needle and passed through a 100‐μm filter. To release cells from splenic tissues, tissues were minced and homogenized by applying gentle pressure with a syringe tip while kept moist with 2% FCS in PBS throughout the procedure and the cell suspension was filtered through a 100‐μm filter and red cells lysed using ammonium chloride (159.65mM). One million events were captured with FACSCanto II (BD Biosciences, San Jose, CA, USA) and analysis was performed with FlowJo software (FlowJo, LLC, Ashland, OR, USA).

### Enzyme‐linked immunosorbent assay

Enzyme‐linked immunosorbent assay (ELISA) kits were used to detect murine tartrate‐resistant acid phosphatase 5b (TRAcP5b), procollagen type 1 N propeptide (P1NP) (Immunodiagnostic Systems, UK), RANKL and osteoprotegerin (OPG) (R&D Systems, Minneapolis, MN, USA) in murine sera following the manufacturer's instructions.

### μCT

Formalin‐fixed left femurs and 4th lumbar vertebrae (L_4_) were imaged with the SkyScan 1772 μCT scanner (Bruker, Kontich, Belgium) at a resolution of 4.3 μm, 0.5 mm aluminum filter, 50 kV voltage and 200 μA tube current. Images were captured every 0.4 degrees through 360 degrees and were reconstructed and analyzed using NRecon software (SkyScan; Bruker, Kontich, Belgium). Regions analyzed in femurs and L_4_ vertebrae have been published.^(^ ^24^ ^)^ Bone structural parameters and nomenclature were utilized according to standardized guidelines.^(^ ^26^ ^)^ Three‐dimensional reconstructed images of femurs and vertebrae were generated using Drishti imaging software version 2.4 (ANU, Canberra, Australia).

### Bone quantitative histomorphometry

Mice were given 250 μL subcutaneous injections of calcein 6 and 2 days prior to cull to label mineralising bone. After euthanasia, right femurs were fixed in 4% paraformaldehyde and processed undecalcified to methylmethacrylate (MMA) resin and were cut at a thickness of 7 μm. Trabecular and endocortical mineral apposition rate (MAR, μm/day), mineralising surface (MS/BS, %) and bone formation rates (BFR/BS, μm^3^/μm^2^/day) were calculated using the double calcein labels measured on trabecular and endocortical bone surfaces.

Paraffin histology was utilized to identify osteoclasts. Left femurs, after μCT analysis, were decalcified in 0.34M ethylenediamine tetraacetic acid (EDTA) (pH 8.0) and were processed for paraffin and 3‐μm sections were cut. To identify osteoclasts, paraffin femur sections were stained for tartrate‐resistant acid phosphatase (TRAP), and staining was performed as described.^(^ ^24^, ^27^ ^)^ The number of TRAP‐positive osteoclasts sitting on the bone surface (N.Oc/BS.Pm) were counted, and osteoclast surface (Oc.S/BS.Pm) was calculated as a percentage of bone surface covered by multinucleated TRAP‐positive osteoclasts.

All resin and paraffin histomorphometry measurements were completed using the Osteomeasure bone histomorphometry software version 3.2.1.8 (OsteoMetrics, Decatur, GA, USA). Using 10 times objective, measurements began at a distance of 0.5 mm from the last chondrocyte of the growth plate. A 2 mm sample length on both trabecular and posterior endocortical surfaces from this offset were used to quantify both mineralization and osteoclast parameters on both trabecular and endocortical bone surfaces. The structural, dynamic, and cellular parameters were calculated and expressed according to the standardized nomenclature.^(^ ^28^ ^)^

### Compression testing of L_4_ vertebrae

After μCT analysis, L_4_ vertebrae were warmed to room temperature (RT) and hydration was maintained with PBS. The vertebral processes were removed prior to testing. Samples were mechanically tested by compression until failure on an Instron 5966 (Instron, Inc., Grove City, PA, USA), and data was collected using BlueHill 3 software version 3 (Instron). Compression testing was performed at 3 mm/min until breaking with a 100‐N load cell. Load displacement curved were plotted and the maximum load to first failure was calculated.

### Statistical analysis

All results were analyzed using Prism software (GraphPad Software, Inc., La Jolla, CA, USA). One‐way analysis of variance (ANOVA) and multiple comparisons were performed using Tukey's correction. Unpaired *t* tests were performed when comparing two populations. All data are expressed as mean with error bars representing standard deviation. The *p* values less than 0.05 were considered statistically significant.

## Results

### Anti‐LRP6 antibody improved bone structure in naïve mice

Treatment of naïve C57BLKalwRij mice with twice‐weekly anti‐LRP6 antibody treatment (Fig. 1*A*) for 7 and 14 days significantly improved trabecular bone structure as determined by μCT analysis (Fig. 1*B*–*D*). Increases in trabecular bone volume in femurs were primarily attributed to elevations in trabecular number at both timepoints (Fig. 1*C*i, ii); while trabecular bone volume changes in lumbar vertebrae were due mostly to changes in trabecular thickness with anti‐LRP6 treatment (Fig. 1*D*i, iii). Anti‐LRP6 antibody treatment had limited impact on cortical bone volume and thickness in femurs and lumbar vertebrae after 7 days of treatment in naïve mice (Table 1). A modest increase in cortical bone volume and thickness in femurs was noted at day 14 (Table 1, *p* = 0.006 and *p* = 0.029, respectively); however, this was not shown in lumbar vertebrae in these mice at the same time point. Nonetheless, consistent with previous studies this improvement in trabecular bone structure in both femurs and lumbar vertebrae demonstrates the ability of anti‐LRP6 antibody to increase bone volume in naïve mice.

**Table 1:** Cortical μCT Results of Femurs and Lumbar Vertebrae (L_4_) Harvested From Naïve Mice Treated With Control or Anti‐LRP6 Antibodies (10 mg/kg) at Days 7 and 14

	Day 7			Day 14		
Parameter	Anti‐LRP6 isotype	Anti‐LRP6 Ab	*p* ^a^	Anti‐LRP6 isotype	Anti‐LRP6 Ab	*p* ^b^
Femur						
Ct.BV (mm^3^)	0.693 ± 0.026	0.687 ± 0.035	0.687	0.565 ± 0.031	0.629 ± 0.037	0.006
Ct.Th (mm)	0.101 ± 0.004	0.098 ± 0.001	0.132	0.078 ± 0.004	0.083 ± 0.004	0.029
L_4_						
Ct.BV (mm^3^)	0.347 ± 0.041	0.358 ± 0.034	0.53	0.337 ± 0.033	0.38 ± 0.04	0.06
Ct.Th (mm)	0.072 ± 0.004	0.072 ± 0.002	0.97	0.065 ± 0.001	0.067 ± 0.003	0.26

*Note*: Results presented as mean ± SD.

Abbreviation: Ab = antibody; Ct.BV = cortical bone volume; Ct.Th = cortical thickness.

^a^ Unpaired *t* test between naïve mice treated with anti‐LRP6 isotype versus anti‐LRP6 Ab after 7 days of treatment.

^b^ Unpaired *t* test between naïve mice treated with anti‐LRP6 isotype versus anti‐LRP6 ab after 14 days of treatment.

**Fig. 2:**
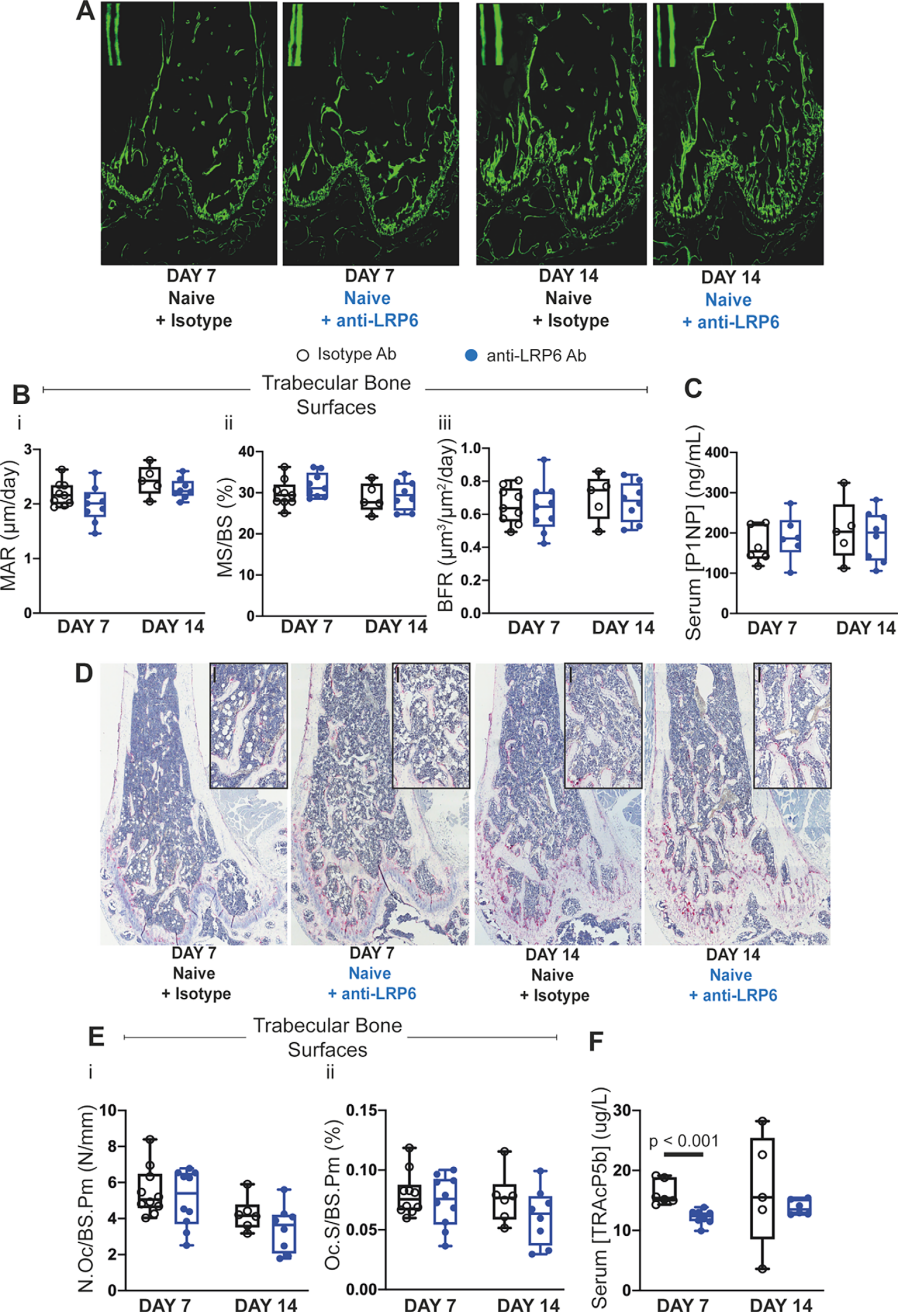
Anti‐LRP6 antibody increased trabecular bone volume in femurs primarily through reduced bone resorption in naïve mice. (*A*) Representative calcein double‐labeled sections of femurs from each treatment group (isotype or anti‐LRP6 Ab) harvested at their respective time points (day 7 or 14). (*B*) (i) MAR (μm/day), (ii) mineralizing surface (MS/BS, %), and (iii) bone formation rate (BFR, μm^3^/μm^2^/day) measured on trabecular surfaces within femurs of naïve mice treated with either isotype or anti‐LRP6 Ab for 7 or 14 days. (*C*) Quantification of serum P1NP (ng/mL) as an indication of systemic expression of bone formation in naïve mice treated with either isotype or anti‐LRP6 Ab for 7 or 14 days. (*D*) Representative images of TRAP‐stained sections of femurs harvested from naïve mice treated with either isotype or anti‐LRP6 Ab for 7 or 14 days. Small black bars represent 100 μm. (*E*) Quantification of (i) number of osteoclasts (N.Oc/BS.Pm, N/mm) and (ii) osteoclast surface (Oc.S/BS.Pm, %) per trabecular bone surfaces in naïve mice treated with either isotype or anti‐LRP6 Ab for 7 or 14 days. (*F*) Quantification of serum TRAcP5b (μg/L) as an indication of systemic expression of bone resorption in naïve mice treated with either isotype or anti‐LRP6 Ab for 7 or 14 days. Results plotted as mean ± SD. Ab = antibody; MAR = mineral apposition rate.

To investigate osteoblast function, we injected mice with calcein 6 and 2 days before the mice were euthanized to label mineralizing surfaces (Fig. 2*A*). As shown in Fig. 2*B*i–iii, anti‐LRP6 treatment did not impact bone formation parameters on trabecular bone surfaces after 7 and 14 days of treatment. This was also reflected on endocortical bone surfaces (data not shown). This observation was confirmed with serum studies of P1NP, with no change after 7 or 14 days of anti‐LRP6 treatment in naïve mice compared to their controls (Fig. 2*C*).

The number of TRAP+ osteoclasts per trabecular bone surface was not shown to be statistically altered in naïve mice after 7 and 14 days of anti‐LRP6 antibody treatment (Fig. 2*D*, *E*i, ii); which was also demonstrated on the endosteal surface (data not shown). Interestingly, serum analysis of TRAcP5b demonstrated significant reductions in systemic expression of bone resorption in naïve mice treated with anti‐LRP6 antibody for 7 days (Fig. 2*F*, *p* < 0.001); however, this reduction was not shown after 14 days of treatment. Additionally, systemic expression of OPG within these mice was elevated after 7 days of anti‐LRP6 treatment (Fig. S1*A*i, *p* < 0.05), with little impact on systemic RANKL levels and the ratio of RANKL/OPG at this timepoint (Fig. S1 *B*, *C*). Neither OPG nor RANKL levels were altered after 14 days of anti‐LRP6 treatment within naïve mice. Thus far, serum studies suggest that bone structural improvements with anti‐LRP6 antibody treatment after 7 and 14 days were primarily achieved through an anti‐resorptive mechanism rather than enhanced bone anabolism.

### Anti‐LRP6 antibody prevented myeloma‐induced bone disease and improved resistance to fracture

**Fig. 3:**
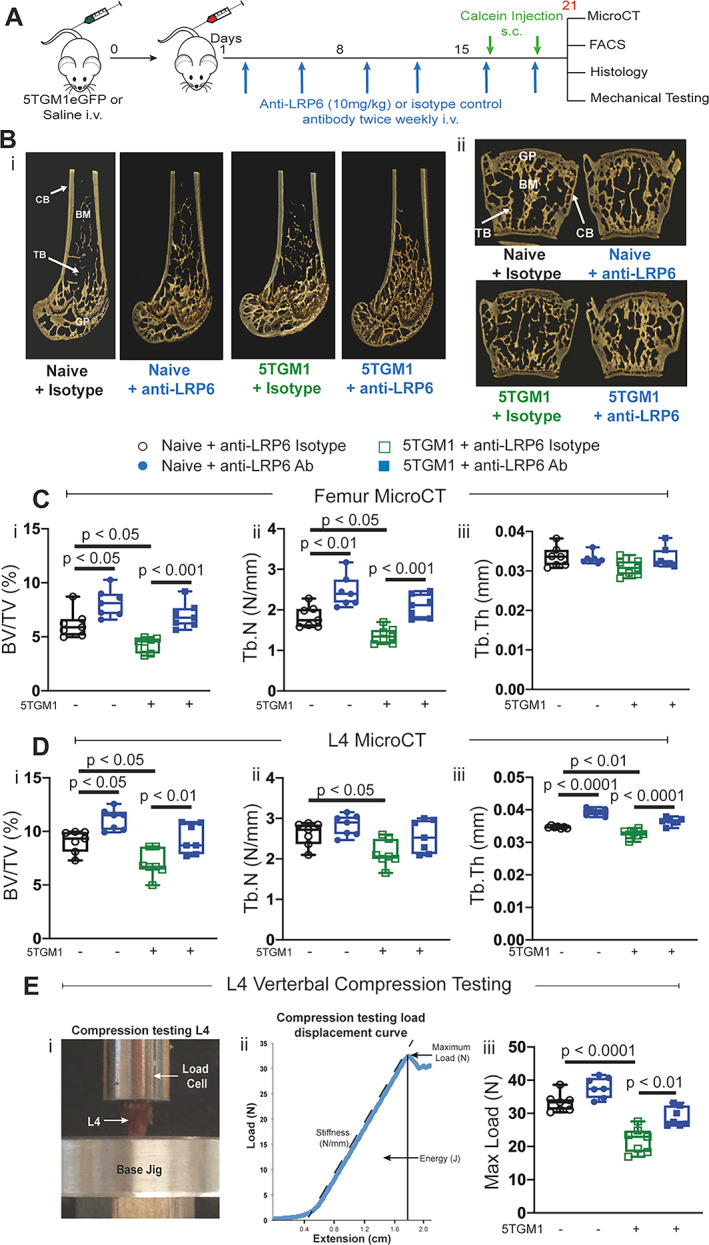
Anti‐LRP6 antibody prevented trabecular bone loss and improved resistance to vertebral compression fractures in 5TGM1‐bearing mice. (*A*) Experimental design and timeline, including antibody treatment duration, fluorochrome labels, and time points where bone tissue were harvested. (*B*) 3D representative images of femurs (i) and L_4_ lumbar vertebrae (ii) harvested from naïve and 5TGM1‐bearing mice treated with either isotype or anti‐LRP6 Ab for 21 days. (*C*) μCT‐derived trabecular bone volume fraction (i, BV/TV, %), trabecular number (ii, Tb.N, N/mm), and trabecular thickness (iii, Tb.Th, mm) in the distal femoral metaphysis in all respective treatment groups. (*D*) μCT‐derived (i) trabecular BV/TV (%), (ii) Tb.N (N/mm), and (iii) Tb.Th (mm) within L_4_ lumbar vertebrae of each respective treatment groups. (*E*) (i) Image of experimental design setup for compression testing of L_4_ lumbar vertebrae, (ii) compression testing load displacement curve for deriving maximum load, (iii) maximum load to failure (Max Load, N) of L_4_ lumbar vertebrae from each respective treatment group. Results plotted as mean ± SD. Ab = antibody; BM = bone marrow; CB = cortical bone; GP = growth plate; i.v. = intravenous; s.c. = subcutaneous; TB = trabecular bone.

As illustrated in Fig. 3*A*, we treated both naïve (non‐tumor‐bearing) mice and mice bearing murine 5TGM1 myeloma cells with anti‐LRP6 antibody twice weekly and measured bone structural changes by μCT analysis (Fig. 3*B*–*D*). As demonstrated at days 7 and 14, naïve mice treated with anti‐LRP6 for 21 days demonstrated increased trabecular bone volume and trabecular number in femurs compared to their controls (Fig. 3*C*i, ii, *p* < 0.05 and *p* < 0.01, respectively), while trabecular bone volume and trabecular thickness were elevated in lumbar vertebrae in these mice (Fig. 3*D*i, iii, *p* < 0.05 and *p* < 0.0001, respectively). Injection of C57BLKalwRij mice with 5TGM1eGFP murine myeloma cells led to significant reductions in trabecular bone volume and trabecular number in femurs (Fig. 3*C*i, ii, *p* < 0.05). μCT of lumbar vertebrae, a common site of fracture in MM patients, also demonstrated significant reductions in trabecular bone parameters in 5TGM1‐bearing mice (Fig. 3*D*i–iii, *p* < 0.05 and *p* < 0.01 for all three parameters). Importantly, this loss in trabecular bone volume was prevented with anti‐LRP6 antibody treatment in 5TGM1‐bearing mice compared to their controls in both femurs (Fig. 3*C*i, ii, *p* < 0.001) and L_4_ lumbar vertebrae (Fig. 3*D*i, iii, *p* < 0.01 and *p* < 0.0001, respectively). Cortical bone structure was not significantly altered in response to anti‐LRP6 antibody treatment in naïve or 5TGM1‐bearing mice (Table 2).

**Table 2:** Cortical μCT Results of Femurs and L_4_ Vertebrae From Naïve and Myeloma‐Bearing (5TGM1eGFP) Mice Treated With Anti‐LRP6 Isotype or Anti‐LRP6 Antibodies (10 mg/kg) for 21 Days (Experiment 1)

	Naive		5TGM1eGFP	
Parameter	Anti‐LRP6 isotype	Anti‐LRP6 Ab	Anti‐LRP6 isotype	Anti‐LRP6 Ab
Femur				
Ct.BV (mm^3^)	0.486 ± 0.019^a^	0.489 ± 0.023	0.459 ± 0.047	0.454 ± 0.042
Ct.Th (mm)	0.077 ± 0.002	0.076 ± 0.004	0.08 ± 0.005	0.077 ± 0.006
L_4_				
Ct.BV (mm^3^)	0.351 ± 0.026	0.361 ± 0.045	0.315 ± 0.045	0.316 ± 0.059
Ct.Th (mm)	0.062 ± 0.001	0.064 ± 0.001	0.06 ± 0.004	0.062 ± 0.003

*Note*: Results presented as mean ± SD.

Abbreviation: Ab = antibody; Ct.BV = cortical bone volume; Ct.Th = cortical thickness.

^a^ *p* < 0.05 between naïve mice treated with anti‐LRP6 isotype versus anti‐LRP6 Ab.

Biomechanical compression testing of vertebrae (Fig. 3*E*i, ii) demonstrated that reductions in BV/TV in L_4_ vertebrae of 5TGM1‐bearing mice reduced their maximum load to failure (*p* < 0.0001, Fig. 3*E*iii). Importantly, anti‐LRP6 antibody treatment prevented reductions in bone strength in 5TGM1‐bearing mice as a result of protection against MM‐induced BV/TV loss (*p* < 0.01, Fig. 3*E*iii), returning the maximum load to failure to naïve mouse isotype levels.

These bone structural changes in response to anti‐LRP6 antibody were replicated in a separate cohort of C57BLKalwRij female mice (results from the second experiment in Fig. S2 and Table 3), to ensure robustness in the experimental approach. These studies confirm the potential for anti‐LRP6 as a therapeutic candidate to address cancer‐induced skeletal related events such as osteolytic lesions.

**Table 3:** Cortical μCT Results of Femurs and L_4_ Vertebrae From Naïve and Myeloma‐Bearing (5TGM1eGFP) Mice Treated With Control or Anti‐LRP6 Antibodies (10 mg/kg) for 21 Days (Experiment 2)

	Naive		5TGM1eGFP	
Parameter	Anti‐LRP6 isotype	Anti‐LRP6 Ab	Anti‐LRP6 isotype	Anti‐LRP6 Ab
Femur				
Ct.BV (mm^3^)	0.545 ± 0.038^a,c^	0.596 ± 0.032^d,f^	0.483 ± 0.038^h^	0.566 ± 0.015
Ct.Th (mm)	0.081 ± 0.004^b^	0.088 ± 0.002^e,g^	0.078 ± 0.004^i^	0.084 ± 0.003
L_4_				
Ct.BV (mm^3^)	0.302 ± 0.036^j,l^	0.357 ± 0.01^n^	0.284 ± 0.044^p^	0.376 ± 0.034
Ct.Th (mm)	0.057 ± 0.005^k,m^	0.064 ± 0.00 ^o^	0.056 ± 0.004^q^	0.064 ± 0.002

*Note*: Results presented as mean ± SD.

Abbreviation: Ab = antibody; Ct.BV = cortical bone volume; Ct.Th = cortical thickness.

^a^ *p* < 0.05, ^b^ *p* < 0.01 compared to Naïve + Anti‐LRP6 Ab mice; ^i^ *p* < 0.05 compared between 5TGM1 anti‐LRP6 isotype and 5TGM1 anti‐LRP6 Ab mice; ^c^ *p* < 0.01 between 5TGM1 + Anti‐LRP6 Isotype mice; ^d,e^ *p* < 0.001 compared to 5TGM1 + Anti‐LRP6 Isotype mice; ^h^ *p* < 0.001 compared to 5TGM1 + Anti‐LRP6 Ab mice; ^f,g^ *p* < 0.05 compared to 5TGM1 + Anti‐LRP6 Ab mice; ^j^ *p* < 0.01, ^k^ *p* < 0.02 compared to Naïve + Anti‐LRP6 Ab mice; ^l,m^ *p* < 0.01 compared to 5TGM1 + Anti‐LRP6 Ab mice; ^n,o^ *p* < 0.01 compared to 5TGM1 + Anti‐LRP6 Isotype mice; ^p,q^ *p* < 0.001 compared to 5TGM1 + Anti‐LRP6 Ab mice.

**Fig. 4:**
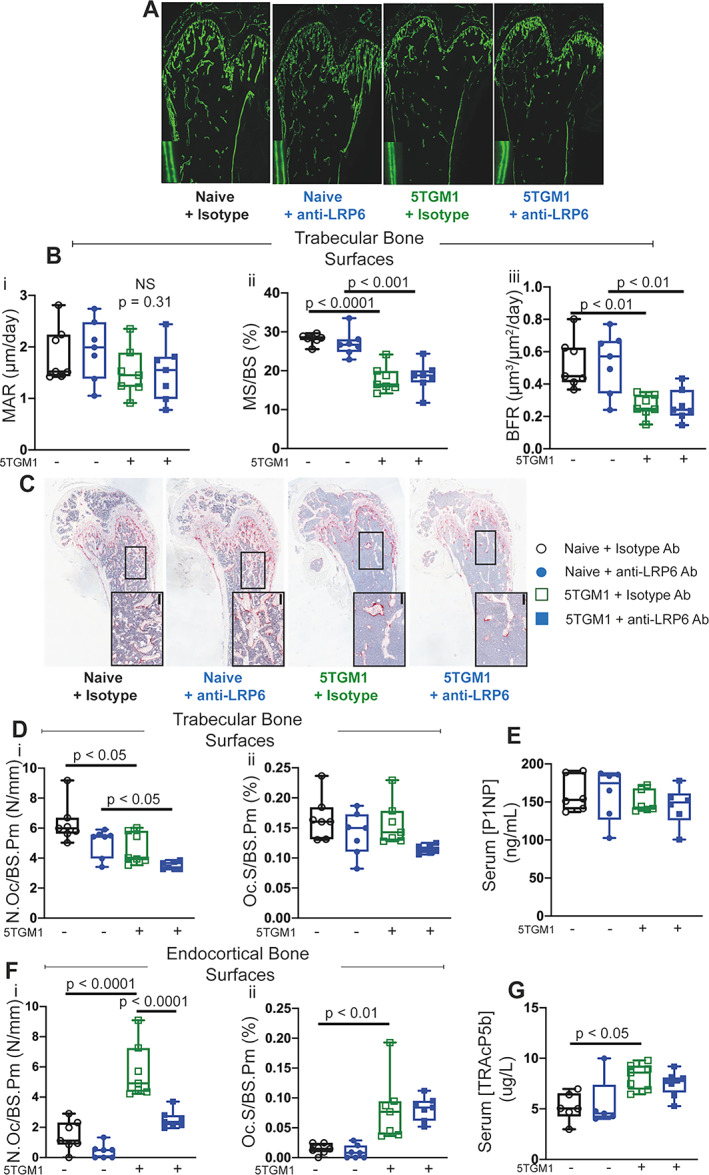
Anti‐LRP6 antibody prevented bone loss primarily through reduced bone resorption in 5TGM1‐bearing mice. (*A*) Representative calcein double‐labeled sections of femurs from naïve or 5TGM1‐bearing mice treated with either isotype or anti‐LRP6 Ab for 21 days. (*B*) (i) Mineral apposition rate (MAR, μm/day), (ii) mineralizing surface (MS/BS, %), and (iii) bone formation rate (BFR, μm^3^/μm^2^/day) measured on trabecular surfaces within femurs of naïve or 5TGM1‐bearing mice treated with either isotype or anti‐LRP6 Ab for 21 days. (C) Representative images of TRAP‐stained sections of femurs from naïve or 5TGM1‐bearing mice treated with either isotype or anti‐LRP6 Ab for 21 days. Small black bars represent 100 μm. (*D*) Quantification of (i) number of osteoclasts (N.Oc/BS.Pm, N/mm) and (ii) osteoclast surface (Oc.S/BS.Pm, %) per trabecular bone surfaces in naïve or 5TGM1‐bearing mice treated with either isotype or anti‐LRP6 Ab for 21 days. (*E*) Quantification of serum P1NP (ng/mL) as an indication of systemic expression of bone formation in naïve and 5TGM1‐bearing mice treated with either isotype or anti‐LRP6 Ab for 21 days. (*F*) Quantification of (i) number of osteoclasts (N.Oc/BS.Pm, N/mm) and (ii) osteoclast surface (Oc.S/BS.Pm, %) per endocortical bone surfaces in naïve mice treated with either isotype or anti‐LRP6 Ab for 21 days. (*G*) Quantification of serum TRAcP5b (μg/L) as an indication of systemic expression of bone resorption in naïve and 5TGM1‐bearing mice treated with either isotype or anti‐LRP6 Ab for 21 days. Results plotted as mean ± SD. Ab = antibody; NS = not significant.

As expected, MAR and consequently BFR were significantly reduced in 5TGM1‐bearing mice compared to their naïve controls on both trabecular (Fig. 4*A*, *B*i‐iii) and endocortical bone surfaces in femurs (data not shown); an outcome common in myeloma patients. Consistent with results shown in naïve mice at the earlier timepoints at day 7 and 14, all bone formation parameters were not elevated with anti‐LRP6 antibody treatment in either naïve or 5TGM1‐bearing mice, which was confirmed with serum analysis of P1NP (Fig. 4*E*).

Osteoclast number and surfaces were significantly elevated in mice bearing 5TGM1 cells on endocortical bone surfaces (Fig. 4*F*, *p* < 0.0001 and *p* < 0.01, respectively). This was confirmed with elevations in circulating levels of TRAcP5b in 5TGM1‐bearing mice treated with the isotype compared to their naïve controls (Fig. 4*G*, *p* < 0.05). Importantly, treatment with anti‐LRP6 antibody significantly reduced the abundance of TRAP+ osteoclasts on endocortical bone surfaces in 5TGM1‐bearing mice (Fig. 4*C*, *F*i, ii, *p* < 0.0001), but not in naïve mice. Osteoclast parameters on trabecular bone surfaces were not increased in the 5TGM1‐bearing mice, nor were they reduced with anti‐LRP6 antibody compared to Isotype control (Fig. 4*D*i, ii). Serum TRAcP5b was significantly elevated in mice bearing 5TGM1 tumors (*p* < 0.05), but was not reduced with anti‐LRP6 treatment (Fig. 4*G*). Overall, these results demonstrate that anti‐LRP6 antibody prevents exacerbated myeloma‐induced bone resorption.

### Combination treatment improves bone structure and strength compared to single treatment in myeloma‐bearing mice

We combined the novel anti‐LRP6 agent with a well‐known inhibitor against the soluble antagonist DKK1, anti‐DKK1, to demonstrate whether this novel combination strategy provides greater protection against bone loss in the context of MM compared to single treatment approaches. First, we confirmed that anti‐DKK1 alone prevented both trabecular and cortical bone loss in 5TGM1‐bearing mice. This protection was demonstrated in both femurs and L_4_ lumbar vertebrae as illustrated in Fig. S3; and this protection in the myeloma setting has been shown elsewhere.^(^ ^22^, ^23^ ^)^ Trabecular BV/TV and Tb.N parameters were almost doubled in naïve mice treated with the anti‐LRP6/DKK1 antibody combination compared to their controls who received the anti‐LRP6/DKK1 isotype combination strategy (Fig. S4*A*i,ii, *p* < 0.001 for both). Trabecular thickness was also elevated with the combination antibody strategy (Fig. S4*A*ii, *p* < 0.01). Importantly, this bone gain with the combination strategy in naïve mice was a result of elevated MAR and BFR parameters on trabecular bone surfaces (Fig. S4*B*i,iii).

**Fig. 5:**
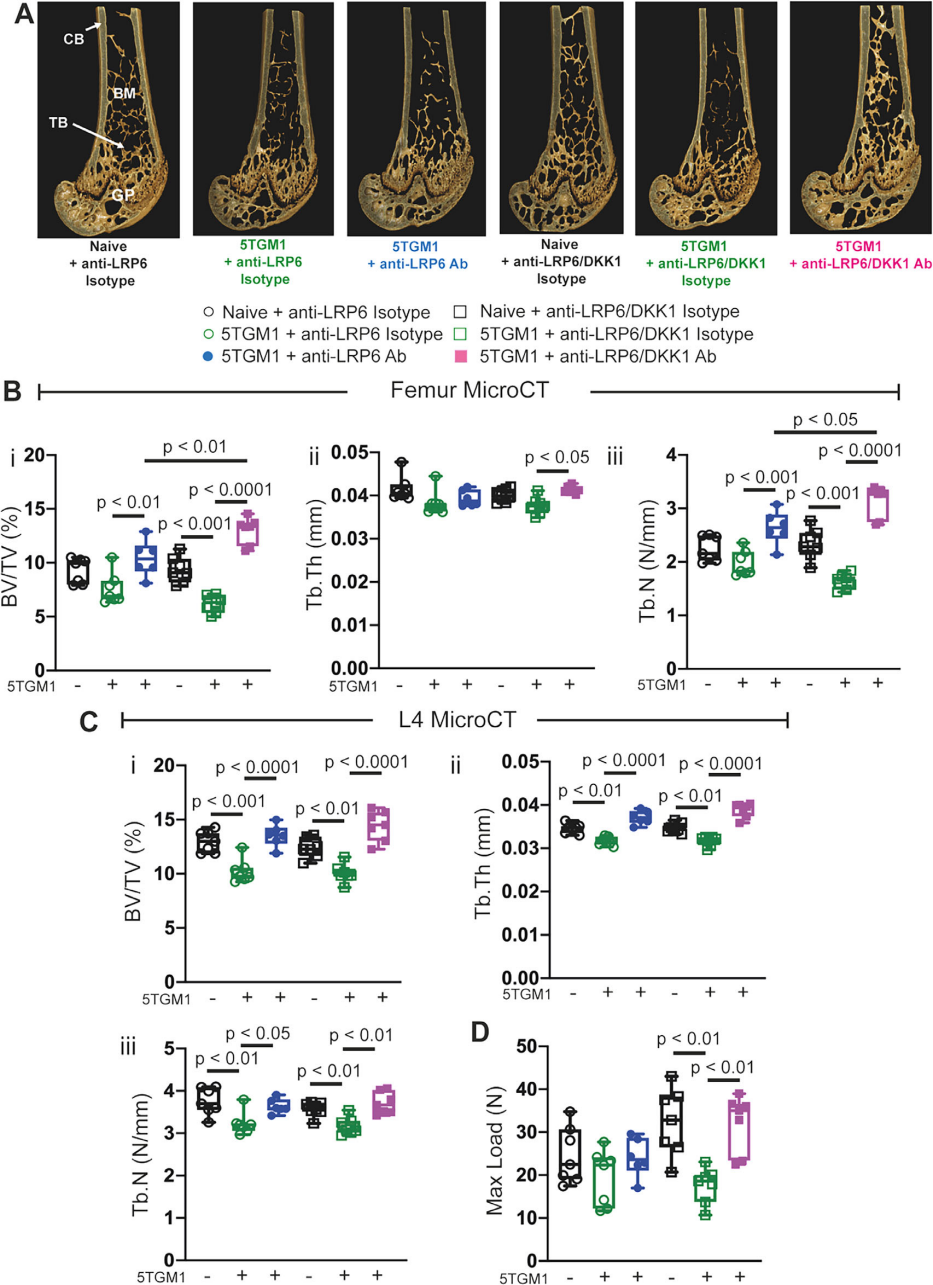
Anti‐LRP6/DKK1 combination strategy provided superior protection against 5TGM1‐induced bone loss and vertebral bone strength compared to single treatment. (*A*) 3D representative images of femurs from naïve or 5TGM1‐bearing mice treated with either anti‐LRP6 Ab alone, anti‐LRP6/DKK1 Ab combination, or their respective isotypes for 21 days. (*B*) μCT‐derived (i) trabecular bone volume fraction (BV/TV, %), (ii) trabecular thickness (Tb.Th, mm), and (iii) trabecular number (Tb.N, N/mm) in the distal femoral metaphysis in all respective treatment groups at day 21. (*C*) μCT‐derived (i) trabecular BV/TV (%), (ii) Tb.Th (mm), and (iii) Tb.N (N/mm) in lumbar L_4_ vertebrae from each respective treatment groups at day 21. (*D*) Maximum load to failure (Max Load, N) of L_4_ lumbar vertebrae from each respective treatment group. Results plotted as mean ± SD. Ab = antibody; BM = bone marrow; CB = cortical bone; GP = growth plate; i.v. = intravenous; s.c. = subcutaneous; TB = trabecular bone.

When we utilized this anti‐LRP6/DKK1 combination approach and compared it to anti‐LRP6 alone in the myeloma setting (Fig. 5*A*), we saw that the combination approach (demonstrated by pink squares in Fig. 5*B*–*D*) more than doubled trabecular BV/TV in 5TGM1‐bearing mice (Fig. 5*B*i, *p* < 0.0001). This significant elevation with the combination strategy was also greater than anti‐LRP6 alone (Fig. 5*B*i, *p* < 0.01). Greater protection against trabecular bone with the anti‐LRP6/DKK1 combination strategy compared to anti‐LRP6 alone was attributed to significant changes in trabecular number rather than thickness (Fig. 5*B*iii, ii, respectively). Protection form 5TGM1 induced cortical bone loss was also superior with the anti‐LRP6/DKK1 combination strategy compared to isotype and anti‐LRP6 alone (Table 4). Similar trabecular bone protection was also demonstrated in lumber vertebrae with the combination strategy compared to anti‐LRP6 alone in 5TGM1‐bearing mice showing significant improvements in both Tb.Th and Tb.N (Fig. 5*C*i–iii). When lumbar compression testing was performed, it was interesting to see in this cohort that anti‐LRP6 alone in 5TGM1‐bearing mice did not reach significance in terms of improving bone strength compared to controls (Fig. 5*D*). Of greater importance, the combination strategy significantly improved resistance to fracture in 5TGM1‐bearing mice compared to controls (Fig. 5*D*, *p* < 0.01), bringing their lumbar vertebral strength back within naïve control levels.

**Table 4:** Cortical μCT Results of Femurs and L_4_ Vertebrae From Naïve and Myeloma‐Bearing (5TGM1eGFP) Mice Treated With Control or Anti‐LRP6 Antibodies or Anti‐LRP6/DKK1 Isotype or Antibodies (10 mg/kg)

	Naive		5TGM1eGFP			
Parameter	Anti‐LRP6 isotype	Anti‐LRP6/DKK1 isotype	Anti‐LRP6 isotype	Anti‐LRP6 Ab	Anti‐LRP6/DKK1 isotype	Anti‐LRP6/DKK1 Ab
Femur						
Ct.BV (mm^3^)	0.613 ± 0.038	0.595 ± 0.019	0.491 ± 0.048^a^	0.556 ± 0.022^e^	0.50 ± 0.023^f^	0.626 ± 0.028
Ct.Th (mm)	0.096 ± 0.003	0.093 ± 0.003	0.084 ± 0.003^b,c^	0.088 ± 0.001^d^	0.089 ± 0.003	0.092 ± 0.002
L_4_						
Ct.BV (mm^3^)	0.316 ± 0.022	0.323 ± 0.022	0.293 ± 0.034	0.318 ± 0.042	0.285 ± 0.016^g^	0.356 ± 0.041
Ct.Th (mm)	0.068 ± 0.003	0.066 ± 0.002	0.064 ± 0.003	0.065 ± 0.004^i^	0.062 ± 0.001^h^	0.072 ± 0.003

*Note*: Results presented as mean ± SD.

Abbreviation: Ab = antibody; Ct.BV = cortical bone volume; Ct.Th = cortical thickness.

^a,b^ *p* < 0.05 compared to 5TGM1 + Anti‐LRP6 Ab mice; ^c^ *p* < 0.05 compared to 5TGM1 + Anti‐LRP6/DKK1 Ab mice; ^d^ *p* < 0.05 compared to 5TGM1 + Anti‐LRP6/DKK1 Ab mice; ^e^ *p* < 0.001 compared to 5TGM1 + Anti‐LRP6/DKK1 Ab mice; ^f^ *p* < 0.001 compared to 5TGM1 + Anti‐LRP6/DKK1 Ab mice; ^g,h^ *p* < 0.001 compared to 5TGM1 + Anti‐LRP6/DKK1 Ab mice; ^i^ *p* < 0.05 compared to 5TGM1 + Anti‐LRP6/DKK1 Ab mice.

### Combination therapy provides superior protection from myeloma‐induced bone resorption

**Fig. 6:**
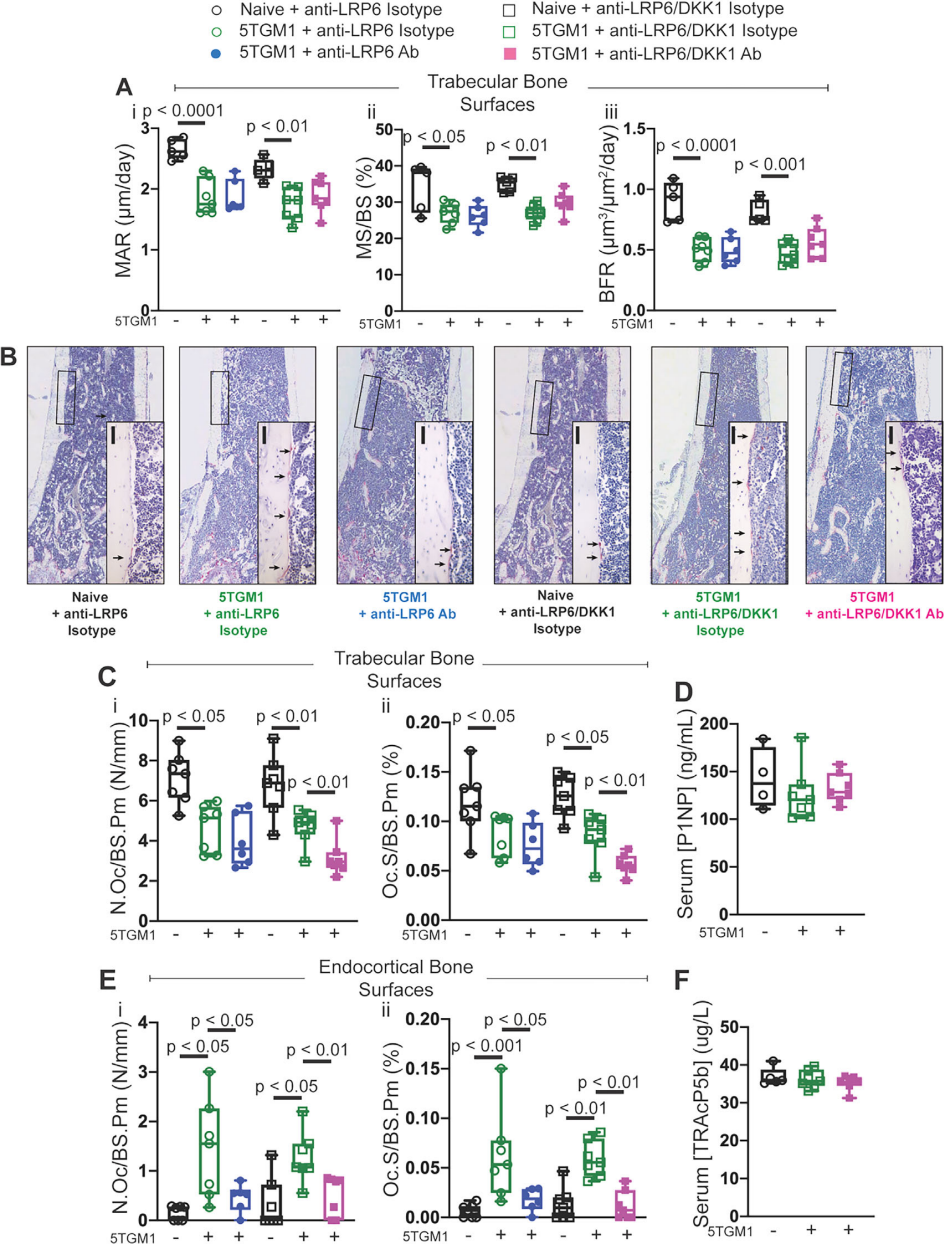
Anti‐LRP6/DKK1 combination strategy reduced bone resorption in 5TGM1‐bearing mice compared to single treatment. (*A*) (i) Mineral apposition rate (MAR, μm/day), (ii) mineralizing surface (MS/BS, %); and (iii) bone formation rate (BFR, μm^3^/μm^2^/day) measured on trabecular surfaces within femurs of naïve or 5TGM1‐bearing mice treated with either anti‐LRP6 Ab alone, anti‐LRP6/DKK1 Ab combination, or their respective isotypes for 21 days. (*B*) Representative images of TRAP‐stained sections of femurs harvested from naïve mice treated with either anti‐LRP6 Ab alone, anti‐LRP6/DKK1 Ab combination, or their respective isotypes for 21 days. Small black scale bars represent 50 μm. (*C*) Quantification of (i) number of osteoclasts (N.Oc/BS.Pm, N/mm) and (ii) osteoclast surface (Oc.S/BS.Pm, %) per trabecular bone surfaces in each respective treatment group after 21 days of treatment. (*D*) Quantification of serum P1NP (ng/mL) as an indication of systemic expression of bone formation in naïve and 5TGM1‐bearing mice treated with either anti‐LRP6/DKK1 Ab combination or its isotype after 21 days of treatment. (*E*) Quantification of (i) number of osteoclasts (N.Oc/BS.Pm, N/mm) and (ii) osteoclast surface (Oc.S/BS.Pm, %) per endocortical bone surfaces in each respective treatment group after 21 days of treatment. (*F*) Quantification of serum TRAcP5b (μg/L) as an indication of systemic expression of bone resorption in naïve and 5TGM1‐bearing mice treated with either anti‐LRP6/DKK1 Ab combination or its isotype after 21 days of treatment. Results plotted as mean ± SD. Ab = antibody.

Bone formation was significantly reduced in 5TGM1‐bearing mice compared to naïve isotype‐treated mice (Fig. 6*A*i–iii). Interestingly, the anti‐LRP6/DKK1 combination strategy did not improve BFR parameters in 5TGM1‐bearing mice compared to anti‐LRP6 alone or control on both trabecular (Fig. 6*A*i–iii) and endocortical (data not shown) bone surfaces. Likewise, serum P1NP levels were not altered in 5TGM1‐bearing mice treated with either the combination strategy or control (Fig. 6*D*). This limited capacity to enhance bone formation was only restricted to myeloma‐bearing mice, as we saw the expected elevation in BFR in naïve age‐matched mice treated with the combination approach (Fig. S4*B*i–iii). Similarly to Fig. 4*F*, reductions in TRAP+ osteoclasts in response to anti‐LRP6 treatment alone reached significance on endocortical bone surfaces (Fig. 6*E*i, ii). Importantly, the combination strategy led to reductions in both osteoclast number and surface in 5TGM1‐bearing mice compared to their 5TGM1‐bearing isotype treated controls on both trabecular and endocortical bone surfaces (Fig. 6*B*, *C*i, ii and Fig. 6*E*i, ii, respectively). Serum TRAcP5b levels on the other hand did not reflect this reduction in bone resorption at the systemic level (Fig. 6*F*). Taken together, these data demonstrate this combination Wnt targeted strategy provided greater protection against myeloma‐induced bone loss and bone strength compared to single treatment strategies.

### Therapeutically stimulating the Wnt/β‐catenin signaling pathway does not alter tumor burden

Controversy exists surrounding the impact of enhanced Wnt signaling and overexpression of β‐catenin on tumor growth and survival in MM and other malignancies.^(^ ^29^, ^30^, ^31^ ^)^ We utilized FACS analysis to examine whether enhanced Wnt signaling with anti‐LRP6 alone or the anti‐LRP6/DKK1 combination strategy exacerbates tumor burden.

**Fig. 7:**
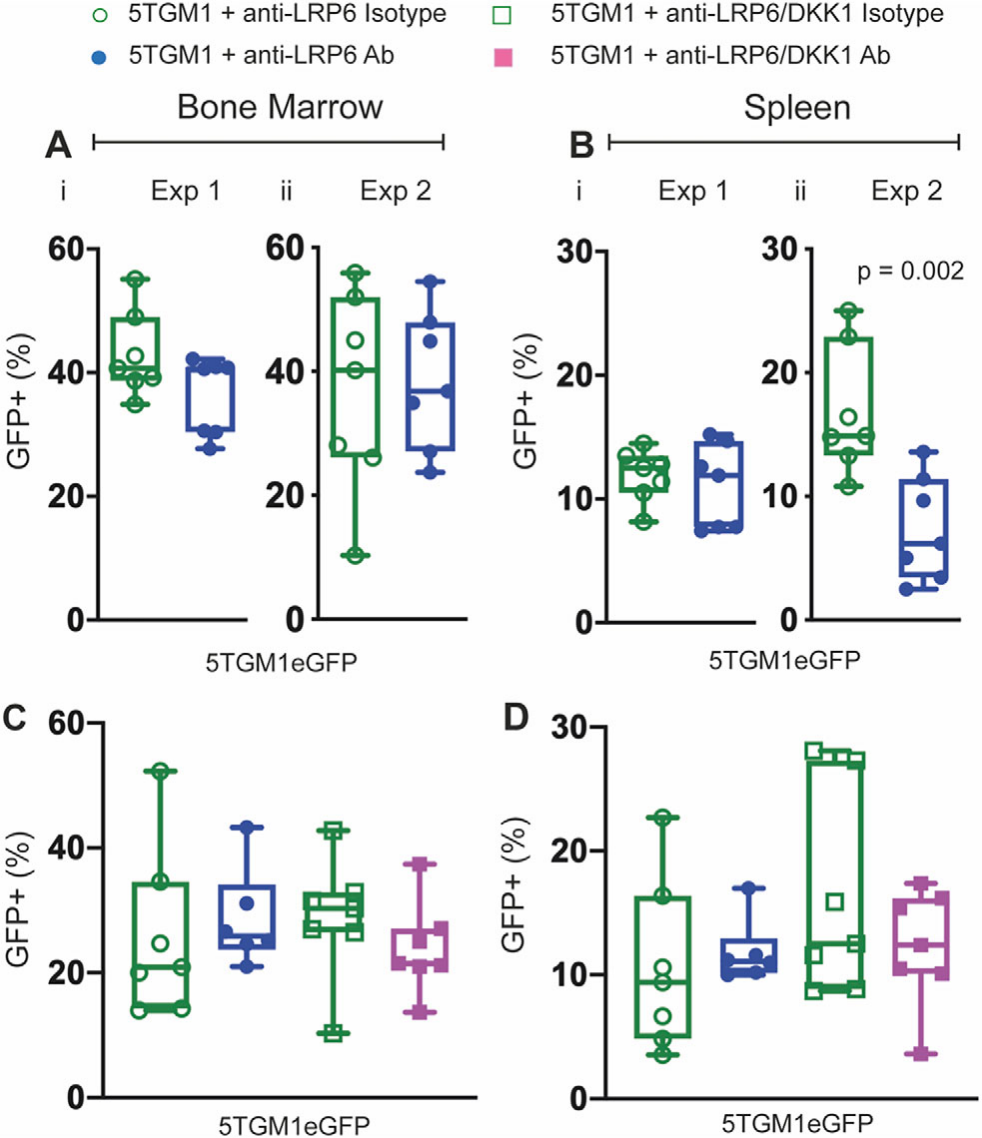
Stimulating Wnt/β‐catenin signaling with Wnt‐targeted agents in single or combination strategies does not exacerbate tumor burden. (*A*,*B*) Proportion of GFP+ myeloma cells within the bone marrow (*A*i–ii) and spleen (*B*i–ii) harvested from 5TGM1‐bearing mice treated with anti‐LRP6 Ab or isotype in two individual experiments. (*C*,*D*) Proportion of GFP+ myeloma cells within the bone marrow and spleen of 5TGM1‐bearing mice treated with either anti‐LRP6 Ab alone, anti‐LRP6/DKK1 Ab combination, or their respective isotypes. Results plotted as mean ± SD. Ab = antibody.

Although there is variation in the raw values between these two independent experiments, FACS analysis validated GFP+ 5TGM1 myeloma cell infiltration within the bone marrow and spleen at the endstage of this model, reported in previous studies.^(^ ^24^, ^27^, ^32^ ^)^ The proportion of GFP+ 5TGM1 cells within the bone marrow was not significantly altered in mice treated with anti‐LRP6 alone (Fig. 7*A*i, ii). Also, the proportion of GFP+ 5TGM1 cells was also not statistically altered in the spleen in the first experiment (Fig. 7*B*i), yet it was shown to be reduced by 56% in response to anti‐LRP6 treatment in experiment 2 compared to control (Fig. 7*B*ii, *p* = 0.002).

When we compared anti‐LRP6 alone with the anti‐LRP6/DKK1 combination strategy, again there was variation in each treatment group, however FACS analysis revealed no changes in tumor burden (Fig. 7*C*, *D*). Although the reduction at the extraskeletal site with anti‐LRP6 alone requires further exploration, these data support the safe use of anti‐LRP6 alone or in combination with anti‐DKK1 to treat patients with myeloma.

## Discussion

Osteolytic bone disease represents a critical and debilitating complication in patients with MM and patients with bone metastasis. Current bone‐targeted treatment strategies that inhibit osteoclasts, including the bisphosphonate zoledronic acid (ZA) and the monoclonal antibody targeting RANKL, denosumab, do not restore bone mass and patients continue to fracture. Therefore, it is imperative to overcome this debilitating disease in MM patients. Therapeutic agents that prevent SOST and DKK1‐inhibition of the Wnt signaling pathway have been previously reported to prevent MM‐induced bone loss and the onset of osteolytic bone lesions.^(^ ^23^, ^24^ ^)^ This investigation, however, is the first to define the cellular mechanisms driving improvements in bone volume with the novel anti‐LRP6 antibody, which potentiates Wnt signaling through binding the LRP6 receptor. We also provide evidence of improved outcomes when delivered with the soluble Wnt‐antagonist targeted therapeutic, anti‐DKK1.

Increased bone mass through elevated Wnt1‐class signaling via the LRP6 receptor was evident in the current investigation, where elevations in trabecular bone volume were apparent after 7, 14, and 21 days of anti‐LRP6 treatment in naïve mice, as shown previously by Chang and colleagues.^(^ ^17^ ^)^ Importantly, treatment of 5TGM1‐bearing mice with this novel anti‐LRP6 antibody prevented myeloma‐induced trabecular bone loss in both femurs and L_4_ lumbar vertebrae, which consequently improved vertebral fracture resistance.

Following work conducted by Florio and colleagues,^(^ ^18^ ^)^ in which a bispecific antibody targeting both DKK1 and SOST provided superior increases in bone mass and fracture repair compared to single treatment approaches, we explored whether a multi‐targeted approach would provide superior outcomes in the setting of MM‐induced bone loss. Unlike the DKK1/SOST dual antibody targeting two soluble antagonists, we chose to target both a membrane bound receptor and a soluble Wnt antagonist, DKK1, which is secreted by myeloma cells to suppress bone formation. The anti‐LRP6/DKK1 combination strategy more than doubled bone volume in 5TGM1‐bearing mice also led to stronger resistance to fracture.

The cellular mechanisms attributed to these improvements in bone structure and strength in response to (1) anti‐LRP6 treatment alone, and (2) the anti‐LRP6/DKK1 combination strategy in both naïve and tumor‐bearing conditions, highlights the complex function of potentiated Wnt/β‐catenin signaling on bone remodeling and skeletal metabolism. Although there are extensive studies focused on characterizing Wnt/β‐catenin signaling within the OB lineage, the effects of Wnt signaling in osteoclasts (OCs) are yet to be fully understood. Selective disruption of the LRP6 receptor within OBs led to deficiencies in trabecular bone architecture in vivo.^(^ ^13^ ^)^ Moreover, deletion of LRP6 within mesenchymal stem cells (MSCs) resulted in severe skeletal complications including significant reductions in both trabecular and cortical bone volume.^(^ ^14^ ^)^ Generation of mice in which Wnt1 was knocked out in early limb mesenchymal cells led to a severe osteopenic phenotype where spontaneous fractures were observed in the limbs, as a result of impaired OB bone formation.^(^ ^9^ ^)^ Additionally, Wnt1 mutations in humans cause severe and early‐onset osteoporosis and osteogenesis imperfecta.^(^ ^33^, ^34^ ^)^ Despite these data, anti‐LRP6 antibody treatment did not lead to overt increases in OB activity, instead the primary mechanism driving increased bone volume was through reduced bone resorption. This unexpected effect affirms previous studies that suggest LRP6, and Wnt/β‐catenin signaling in general, might not solely drive OB differentiation and bone formation,^(^ ^10^, ^35^ ^)^ but rather this pathway also regulates osteoclastogenesis both directly and indirectly.^(^ ^9^, ^36^, ^37^ ^)^ Indeed, the number of OCs was reduced in mice where the LRP6 receptor was deleted in MSCs in vivo^(^ ^14^ ^)^ and Wnt1 mutations have been associated with increased bone resorption.^(^ ^9^ ^)^ LRP5 and LRP6 receptors (among other Wnt receptors) are also expressed on OC progenitor cells and mature OCs; and stimulating Wnt signaling via Wnt3a treatment in OC in vitro cell cultures suppressed OC differentiation.^(^ ^38^ ^)^ Therefore, to explore whether there was an indirect effect of LRP6 treatment on osteoclasts in our study, we examined circulating OPG and sRANKL levels. Although serum OPG levels were increased after 7 days of anti‐LRP6 treatment in naïve mice, RANKL levels were not altered (Fig. S1). In addition, serum TRAcP5b was significantly reduced at the same time point (Fig. S2), but by day 14 of treatment these changes were no longer detected. There observations require further exploration, as they suggest that increased Wnt/β‐catenin signaling may influence components of downstream pathways, for example OPG and RANKL,^(^ ^39^ ^)^ that can modify the activity of other bone cell populations (reviewed by Bodine and Komm^(^ ^40^ ^)^). These alterations in OPG/RANKL signaling may also implicate osteomorphs and osteoclast recycling^(^ ^41^ ^)^ in the mechanism of action of anti‐LRP6, but this was not within the scope of this study.

Although greater modifications to OCs were shown with anti‐LRP6 antibody single treatment, we cannot definitively state that this antibody has limited to no effect on OB function, particularly when we combined anti‐LRP6 with the bone anabolic, anti‐DKK1. In fact, when we treated a small number of naïve mice with this combination strategy for 21 days, we saw elevations in bone volume on trabecular bone surfaces (Fig. S4*A*). Importantly, we also saw the expected elevations in bone formation as demonstrated through calcein fluorescent labelling (Fig. S4*B*). Although, because a direct comparison with anti‐LRP6 alone was not explored here, this increased bone formation could be attributed to the anti‐DKK1 antibody in this combination group. Future studies exploring this combination treatment strategy in naïve mice should be explored to validate this. Despite the fact that changes in bone formation in response to anti‐LRP6/DKK1 combination were not observed within the context of myeloma in 5TGM1‐bearing mice, the anti‐resorptive ability with this combination strategy was far superior when compared to anti‐LRP6 alone. One aspect we did not explore was circulating levels of DKK1 and SOST in both naïve and myeloma‐bearing conditions, and the influence of either single or combination strategies with anti‐LRP6 and anti‐DKK1 on these antagonists. Studies have shown that DKK1 and SOST may exhibit mutual compensatory regulation, where one antagonist is highly expressed when the other is selectively suppressed.^(^ ^42^, ^43^, ^44^ ^)^ The lack of increased bone formation within these mice may have been due to circulating levels of these antagonists (among others) preventing the increases in bone formation we were expecting with the anti‐LRP6/DKK1 combination strategy. Increasing the dosage of either anti‐LRP6, anti‐DKK1 or both of these agents may overcome this suppression. Although changes in OC activity in response to anti‐LRP6 treatment was the primary finding of this study, it would be interesting to explore this novel anti‐LRP6 antibody alone in murine models with osteoblast specific deletion of downstream β‐catenin which leads to low bone mass phenotypes.^(^ ^37^, ^45^ ^)^ This would allow us to determine whether anti‐LRP6 has any direct effect on OC function and differentiation, and whether it has the capacity to restore bone volume within these models independently of osteoblasts.

Nonetheless, the combination of anti‐LRP6 and anti‐DKK1 antibodies provided superior protection against myeloma‐induced bone loss, as well as providing superior protection against myeloma‐induced vertebral fracture compared to the single treatment approach. This highlights the potential for combining anti‐LRP6 with other Wnt‐targeted soluble antagonists, such as the potent bone anabolic anti‐SOST (romosozumab), or even the dual anti‐SOST/DKK1 antibody,^(^ ^18^ ^)^ to provide even greater protection against myeloma‐induced bone disease.

Overall, this is the first study to define the cellular mechanisms driving improvements in bone volume in both naïve and myeloma‐bearing mice treated with the novel LRP6 receptor targeting antibody, anti‐LRP6. Additionally, this is the first study to demonstrate the potential application of combining multiple Wnt‐stimulating therapeutics to prevent bone loss in a murine model of myeloma, targeting both soluble antagonists elevated in myeloma, and receptors of this pathway. Our data also provide new insight into the complex capacity for the canonical Wnt/β‐catenin signaling pathway to regulate bone remodeling and skeletal metabolism, and the importance of exploring all components of this pathway in both naïve and cancer‐induced bone disease models. Taken together, these outcomes will lead to a new era of investigating combination Wnt‐targeted therapies to address the skeletal complications associated with multiple myeloma and other bone destructive cancers.

## Author Contributions

**Marija K. Simic:** Conceptualization; methodology; data curation; formal analysis; writing – original draft; visualization; writing – review and editing; investigation. **Sindhu T. Mohanty:** Methodology; data curation. **Ya Xiao:** Methodology; data curation. **Tegan L. Cheng:** Methodology; data curation. **Victoria E. Taylor:** Data curation; visualization; writing – review and editing. **Olga Charlat:** Resources. **Peter I. Croucher:** Supervision; funding acquisition. **Michelle M. McDonald:** Conceptualization; funding acquisition; writing – review and editing; supervision; methodology.

## Disclosures

The authors declare no conflicts of interest.

### Peer Review

The peer review history for this article is available at https://www.webofscience.com/api/gateway/wos/peer‐review/10.1002/jbmr.4809.

## Supporting information


**Fig. S1.** Circulating levels of OPG and RANKL in response to anti‐LRP6 antibody treatment in naïve mice. Systemic expression of (A) OPG, (B) RANKL and (C) RANKL/OPG in naïve mice treated with anti‐LRP6 Ab or isotype for 7 and 14 days. Results plotted as mean ± SD.
**Fig. S2.** Anti‐LRP6 antibody prevented trabecular bone loss in a separate cohort of 5TGM1‐bearing mice. μCT‐derived (i) trabecular bone volume fraction (BV/TV, %), (ii) trabecular thickness (Tb.Th, mm), and (iii) trabecular number (Tb.N, N/mm) in the distal femoral metaphysis (A) and L4 lumbar vertebrae (B) in all respective groups in repeated 5TGM1‐bearing mice treated with anti‐LRP6 AB alone or isotype for 21 days. Results plotted as mean ± SD.
**Fig. S3.** Anti‐DKK1 antibody prevented trabecular bone loss in 5TGM1‐bearing mice. μCT‐derived (i) trabecular bone volume fraction (BV/TV, %), (ii) trabecular thickness (Tb.Th, mm), (iii), trabecular number (Tb.N, N/mm), (iv) cortical bone volume (Ct.BV, mm^3^) and (v) cortical thickness (Ct.Th, mm) in the distal femoral metaphysis (A) and L4 lumbar vertebrae (B) of naïve and 5TGM1‐bearing mice treated with anti‐DKK1 Ab alone or isotype for 21 days. Results plotted as mean ± SD.
**Fig. S4.** Anti‐LRP6/DKK1 combination strategy led to significant elevations in bone volume in naïve mice. (A) μCT‐derived (i) trabecular bone volume fraction (BV/TV, %), (ii) trabecular thickness (Tb.Th, mm), (iii) trabecular number (Tb.N, N/mm), (iv) cortical bone volume (Ct.BV, mm^3^) and (v) cortical thickness (Ct.Th, mm) in the distal femoral metaphysis. (i) Mineral apposition rate (MAR, μm/day), mineralizing surface (MS/BS, %) and (iii) bone formation rate (BFR, μm^3^/μm^2^/day) measured on trabecular (B) and endocortical bone (C) surfaces within femora of naïve mice treated with anti‐LRP6/DKK1 Ab combination or isotype for 21 days. Results plotted as mean ± SD.

## Data Availability

The data that support the findings of this study are available from the corresponding author upon reasonable request.
