# Supplementary material for: Multi‐Targeting DKK1 and LRP6 Prevents Bone Loss and Improves Fracture Resistance in Multiple Myeloma
Source: J Bone Miner Res. 2023 May 25;38(6):814–28. doi: 10.1002/jbmr.4809 (PMC10947379; doi:10.1002/jbmr.4809)

Multi-targeting DKK1 and LRP6 Prevents Bone Loss and Improves Fracture Resistance in Multiple Myeloma

Marija K. Simic[1,2], Sindhu T. Mohanty[1], Ya Xiao[1], Tegan L. Cheng[3], Victoria E. Taylor[1], Olga Charlat[4], Michael Daley[4], Peter I. Croucher[1,2], Michelle M. McDonald[1,2,5]

1. Skeletal Diseases Program, Garvan Institute of Medical Research, Darlinghurst, NSW Australia
2. St Vincent's Clinical Campus, School of Clinical Medicine, University of New South Wales, Kensington NSW Australia
3. Centre for Children's Bone and Musculoskeletal Health, The Children's Hospital at Westmead, Westmead NSW Australia
4. Novartis Institutes for Biomedical Research, Cambridge MA, USA
5. School of Medical Science, Faculty of Medicine and Health, The University of Sydney, NSW Australia

Corresponding author:
Michelle M. McDonald, Ph.D
Skeletal Diseases Program
Garvan Institute of Medical Research
384 Victoria Street
Darlinghurst NSW 2010
Tel: +612 9295 8247
Email: m.mcdonald@garvan.org.au

Running title: Wnt Stimulation in Myeloma Bone Disease

Keywords: Wnt, LRP6, DKK1, myeloma, bone

SUPPLEMENTARY FIGURE LEGENDS

Supplementary figure 1. Circulating levels of OPG and RANKL in response to anti-LRP6 antibody treatment in naïve mice. Systemic expression of **(A)** OPG, **(B)** RANKL and **(C)** RANKL/OPG in naïve mice treated with anti-LRP6 Ab or isotype for 7 and 14 days. Results plotted as mean ± SD.

Supplementary figure 2. Anti-LRP6 antibody prevented trabecular bone loss in a separate cohort of 5TGM1-bearing mice. MicroCT-derived (i) trabecular bone volume fraction (BV/TV, %), (ii) trabecular thickness (Tb.Th, mm), and (iii) trabecular number (Tb.N, N/mm) in the distal femoral metaphysis **(A)** and L4 lumbar vertebrae **(B)** in all respective groups in repeated 5TGM1-bearing mice treated with anti-LRP6 AB alone or isotype for 21 days. Results plotted as mean ± SD.

Supplementary figure 3. Anti-DKK1 antibody prevented trabecular bone loss in 5TGM1-bearing mice. MicroCT-derived (i) trabecular bone volume fraction (BV/TV, %), (ii) trabecular thickness (Tb.Th, mm), (iii), trabecular number (Tb.N, N/mm), (iv) cortical bone volume (Ct.BV, mm^3) and (v) cortical thickness (Ct.Th, mm) in the distal femoral metaphysis **(A)** and L4 lumbar vertebrae **(B)** of naïve and 5TGM1-bearing mice treated with anti-DKK1 Ab alone or isotype for 21 days. Results plotted as mean ± SD.

Supplementary figure 4. Anti-LRP6/DKK1 combination strategy led to significant elevations in bone volume in naïve mice. (A) MicroCT-derived (i) trabecular bone volume fraction (BV/TV, %), (ii) trabecular thickness (Tb.Th, mm), (iii) trabecular number (Tb.N, N/mm), (iv) cortical bone volume (Ct.BV, mm^3) and (v) cortical thickness (Ct.Th, mm) in the distal femoral metaphysis. (i) Mineral apposition rate (MAR, μm/day), mineralising surface (MS/BS, %) and (iii) bone formation rate (BFR, $\mu m^3/\mu m^2$/day) measured on trabecular **(B)** and endocortical bone **(C)** surfaces within femora of naïve mice treated with anti-LRP6/DKK1 Ab combination or isotype for 21 days. Results plotted as mean ± SD.

Supplementary figure 1.

57 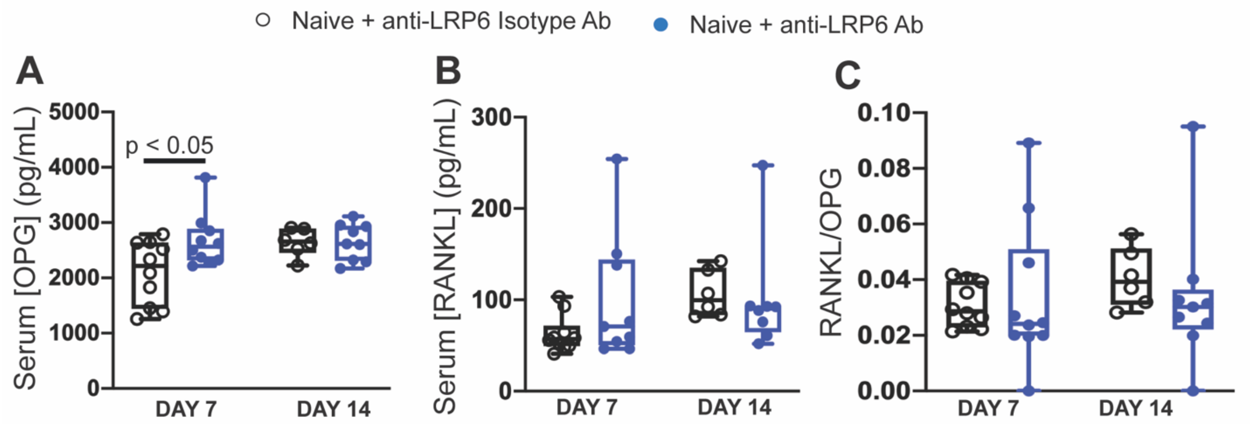

Supplementary figure 2.

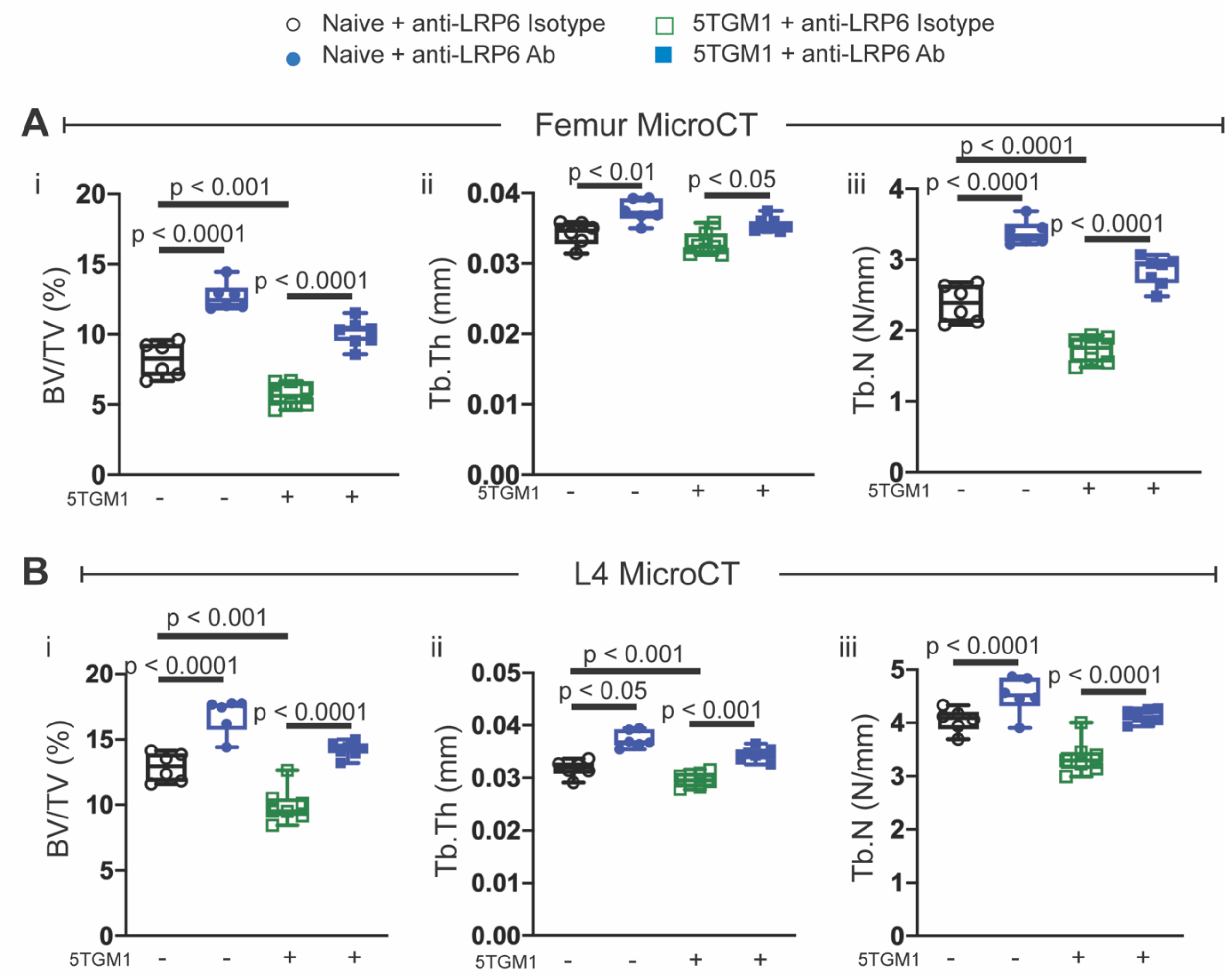

Supplementary figure 3.

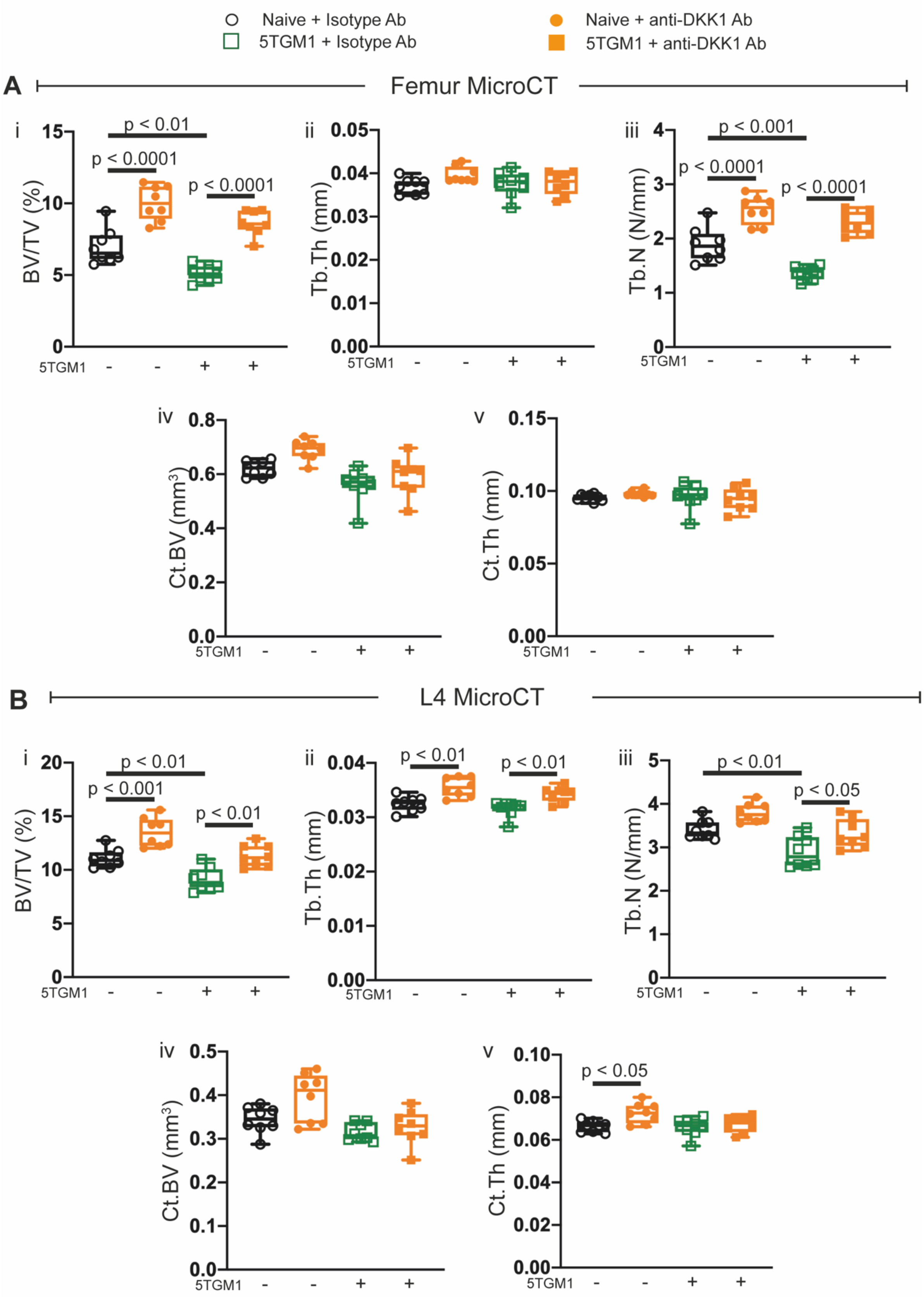

Supplementary figure 4

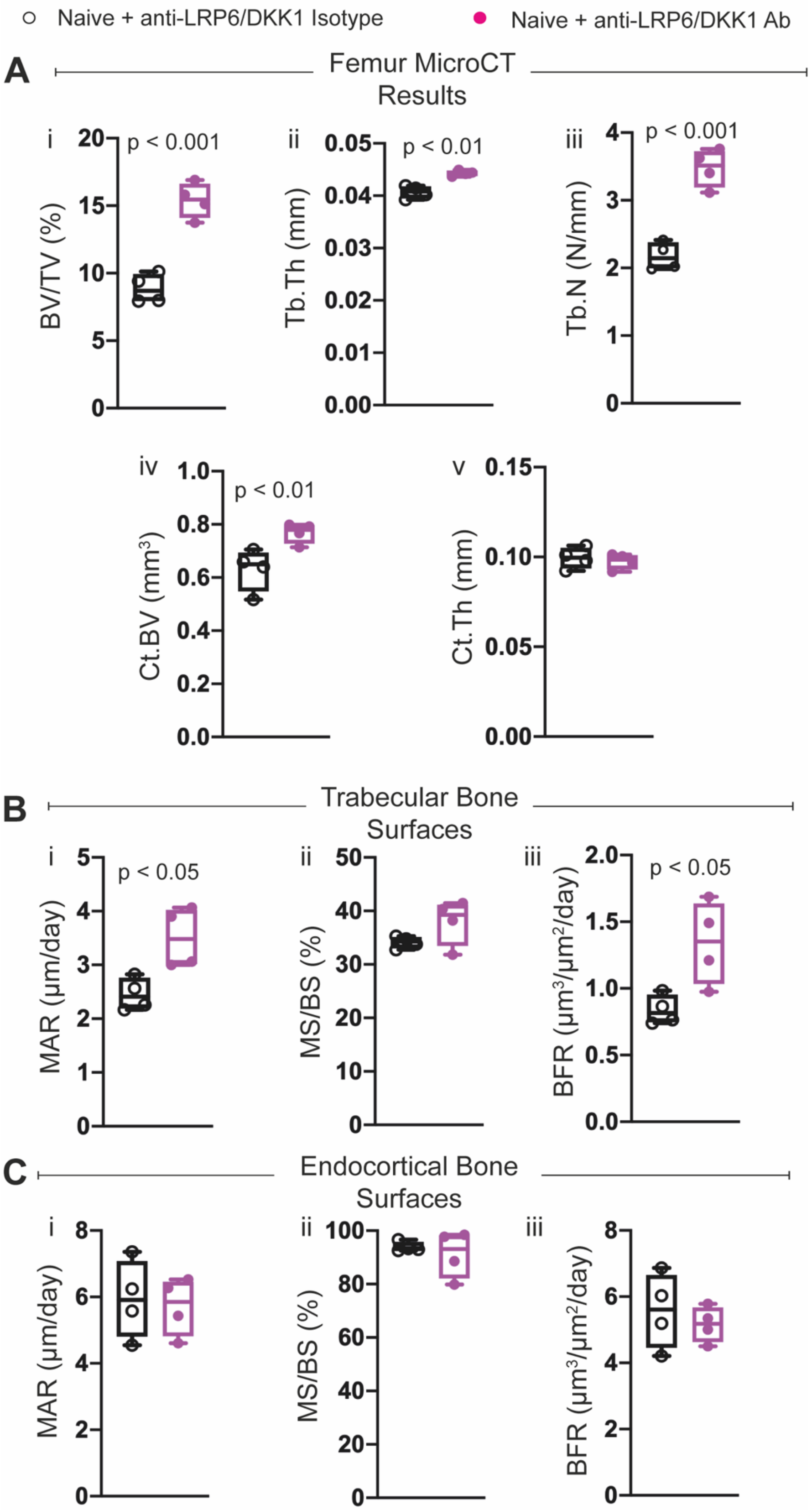

Supplement: Supplementary file 1 — Fig. S1. Circulating levels of OPG and RANKL in response to anti‐LRP6 antibody treatment in naïve mice. Systemic expression of (A) OPG, (B) RANKL and (C) RANKL/OPG in naïve mice treated with anti‐LRP6 Ab or isotype for 7 and 14 days. Results plotted as mean ± SD. Fig. S2. Anti‐LRP6 antibody prevented trabecular bone loss in a separate cohort of 5TGM1‐bearing mice. μCT‐derived (i) trabecular bone volume fraction (BV/TV, %), (ii) trabecular thickness (Tb.Th, mm), and (iii) trabecular number (Tb.N, N/mm) in the distal femoral metaphysis (A) and L4 lumbar vertebrae (B) in all respective groups in repeated 5TGM1‐bearing mice treated with anti‐LRP6 AB alone or isotype for 21 days. Results plotted as mean ± SD. Fig. S3. Anti‐DKK1 antibody prevented trabecular bone loss in 5TGM1‐bearing mice. μCT‐derived (i) trabecular bone volume fraction (BV/TV, %), (ii) trabecular thickness (Tb.Th, mm), (iii), trabecular number (Tb.N, N/mm), (iv) cortical bone volume (Ct.BV, mm3) and (v) cortical thickness (Ct.Th, mm) in the distal femoral metaphysis (A) and L4 lumbar vertebrae (B) of naïve and 5TGM1‐bearing mice treated with anti‐DKK1 Ab alone or isotype for 21 days. Results plotted as mean ± SD. Fig. S4. Anti‐LRP6/DKK1 combination strategy led to significant elevations in bone volume in naïve mice. (A) μCT‐derived (i) trabecular bone volume fraction (BV/TV, %), (ii) trabecular thickness (Tb.Th, mm), (iii) trabecular number (Tb.N, N/mm), (iv) cortical bone volume (Ct.BV, mm3) and (v) cortical thickness (Ct.Th, mm) in the distal femoral metaphysis. (i) Mineral apposition rate (MAR, μm/day), mineralizing surface (MS/BS, %) and (iii) bone formation rate (BFR, μm3/μm2/day) measured on trabecular (B) and endocortical bone (C) surfaces within femora of naïve mice treated with anti‐LRP6/DKK1 Ab combination or isotype for 21 days. Results plotted as mean ± SD. [file JBMR-38-814-s001.pdf]
